# Periprosthetic and Interprosthetic Femoral Fractures: A 20-Year Retrospective Prevalence Analysis Conducted at a Greek Referral Orthopaedic Centre

**DOI:** 10.7759/cureus.78592

**Published:** 2025-02-05

**Authors:** Georgios Paparoidamis, Eustathios Kenanidis, Dimitrios Grammatikopoulos, Michael Potoupnis, Eleftherios Tsiridis

**Affiliations:** 1 Academic Orthopaedic Department, Aristotle University Medical School, General Hospital Papageorgiou, Thessaloniki, GRC

**Keywords:** epidemiological study, periprosthetic femoral fractures, periprosthetic fracture registry, total hip arthroplasty: tha, total knee arthroplasty (tka)

## Abstract

Introduction

The prevalence of periprosthetic femoral fractures (PFFs) in Greece has not been previously documented. This study aims to determine the prevalence of PFFs in a Greek population over the past 20 years, using data from a referral centre.

Methods

A retrospective analysis of PFFs was conducted at a Greek academic orthopaedic department, covering the period from 2004 to 2023. Demographic data, PFF types, treatment methods, time from admission to surgery, length of hospital stay (LOS), and operative times were recorded and compared between the two decades.

Results

The study included 244 patients with PFFs, with a mean age of 78.2 years. Most patients were female (86.5%, p < 0.001). The incidence of PFFs significantly increased between the first and second decades (mean: 9.8 vs. 14.6 cases per year, p = 0.01). This increase was particularly evident in fractures around total knee arthroplasties (TKAs) (p = 0.0027). Treatment choices between internal fixation and revision arthroplasty remained consistent over time for PFFs around total hip arthroplasties (THAs) and TKAs (p > 0.05). However, the LOS and the time from admission to surgery significantly decreased from the first to the second decade (p = 0.001 and p = 0.02), respectively.

Conclusion

This study is the first to document PFFs in a Greek population, showing a notable increase in incidence, higher prevalence among females, consistent treatment methods, and a reduction in the time from admission to surgery and LOS over the past decade.

## Introduction

Fragility fractures of the proximal femur are becoming increasingly prevalent in the population and could be a modern epidemic [1]. The increase in life expectancy and the high prevalence of osteoporosis are the main causes of this phenomenon [1,2]. On the other hand, the number of total hip and knee replacements (total hip arthroplasties (THAs), total knee arthroplasties (TKAs)) and revision surgeries is also rapidly increasing and will continue to grow as the population ages [3]. As a result, a growing number of elderly patients undergoing THA or TKA are at a higher risk of fragility fractures [3].

Periprosthetic fractures (PFs) are fractures that occur in the vicinity of orthopaedic implants, including both replacement and internal fixation devices [2]. Periprosthetic femoral fractures (PFFs) are femoral fractures that are associated with hip or knee prostheses. The management principles and prognosis for PFFs are primarily based on three key factors: the fracture's location, the implant's stability, and the extent of bone loss [4]. Techniques for addressing PFFs include open reduction internal fixation (ORIF) and revision arthroplasty, a more demanding surgical procedure [5]. The management of PFFs is complex, often leading to longer hospital stays and increased postoperative complications, with a significant economic impact on healthcare systems [3,5]. Understanding the distinctive traits and exploring personalised approaches to managing PFFs is essential for optimising outcomes in this patient population [5].

The demographic trend of an ageing population, coupled with the rising number of THAs and TKAs, can contribute to a notable epidemic increase in PFFs [1,2,5]. However, national epidemiological prevalence studies of PFFs are limited [4]. This study aims to highlight, for the first time, the prevalence of PFFs in a Greek population over the past 20 years, providing valuable insights into trends, risk factors, and regional healthcare needs that can inform preventive strategies, resource allocation, and patient management in Greece. Epidemiologic data of the patients, time from admittance to surgery, length of hospital stay (LOS), and surgical treatment methods employed during this period were documented and analysed.

## Materials and methods

The present study was conducted at an academic orthopaedic department of a tertiary hospital after obtaining approval from the institution's scientific board and bioethics committee (171822/2024-16.07.2024). The hospital primarily serves an urban and suburban population, with occasional cases referred from rural areas. Due to the study's retrospective nature, which spanned over two decades ago, informed consent from the participants was decided not to be sought. Data were recorded in the regional Arthroplasty Registry Thessaloniki (ART).

This study is a retrospective analysis aiming to document the prevalence of PFFs at a Greek academic orthopaedic department over 20 years from 2004 to 2023. All PFF cases admitted to the orthopaedic department were identified during this timeframe. Two independent investigators identified patients admitted to the hospital with a diagnosis of PFF by utilising the corresponding ICD-10 code (M96.6, ICD-10-GrM, 2024). The two investigators manually searched all medical records in our department to ensure complete case identification. Data were extracted from the institution’s archived hard copy medical records (before 2012) and digital records from the institution’s electronic database (2012 and thereafter). The identities of the included patients remained confidential throughout the entire process, and personally identifiable information wasn't used in the analysis.

Patients admitted to and treated at our hospital between 2004 and 2023 after suffering a PFF around THA or TKA, between THA and TKA, gamma nail or other femoral internal fixation device were included in the study. All other femoral fractures, including those PFFs with an incomplete or insufficient medical file, were excluded from the study. 

The two independent investigators assessed the preoperative and postoperative X-rays and the medical records of the patients under the supervision of an experienced attending surgeon and collected the following data: 

a) Demographic data and complete medical history.

b) The PFF type that was based on established classification systems. The Vancouver classification was used for PFFs around THA [6], while the Lewis and Rorabeck classification [7] and the Su and Associates’ classification were used for PFFs around TKAs [8]. Additionally, the Vergilius classification was used for PFFs around γ-nails [9], and the Pires classification was used for Interprosthetic PFFs between THA and TKA [10].

c) The treatment method, including conservative treatment, ORIF with plates and screws and/or cerclage wires or a retrograde nailing, revision arthroplasty, revision nail with a longer nail, or a combination of the above.

d) Time from admission to surgery, LOS and operative time.

The PFFs were divided into those that occurred during the first decade (2004-2013) and those that occurred during the second decade (2014-2023). The researchers assessed differences in a) the mean prevalence, b) the mean age and other demographics, c) the PFF types and treatment methods employed, and d) the mean time from admission to surgery and LOS between the two decades.

Statistical analysis

Descriptive statistics summarised the data, with normality assessed using the Shapiro-Wilk test. Two-tailed tests were used for inferential analyses. Independent t-tests analysed age differences across genders and decades, while Wilcoxon rank-sum tests were applied when non-parametric alternatives were needed. Comparisons of treatment methods across prosthesis types utilised both parametric and non-parametric tests. Pearson’s chi-squared test with Yates' correction evaluated the distribution of treatment methods (ORIF vs. revision arthroplasty) across decades. Fisher’s exact test assessed associations between treatment choices and decades for knee fractures. Pearson’s correlation coefficient and logistic regression analysed relationships between patient characteristics and treatment methods. All analyses were conducted using R software (version 2023.12.0+369, RStudio, Posit, Boston, MA), with significance set at p < 0.05.

## Results

Demographics

**Figure 1:**
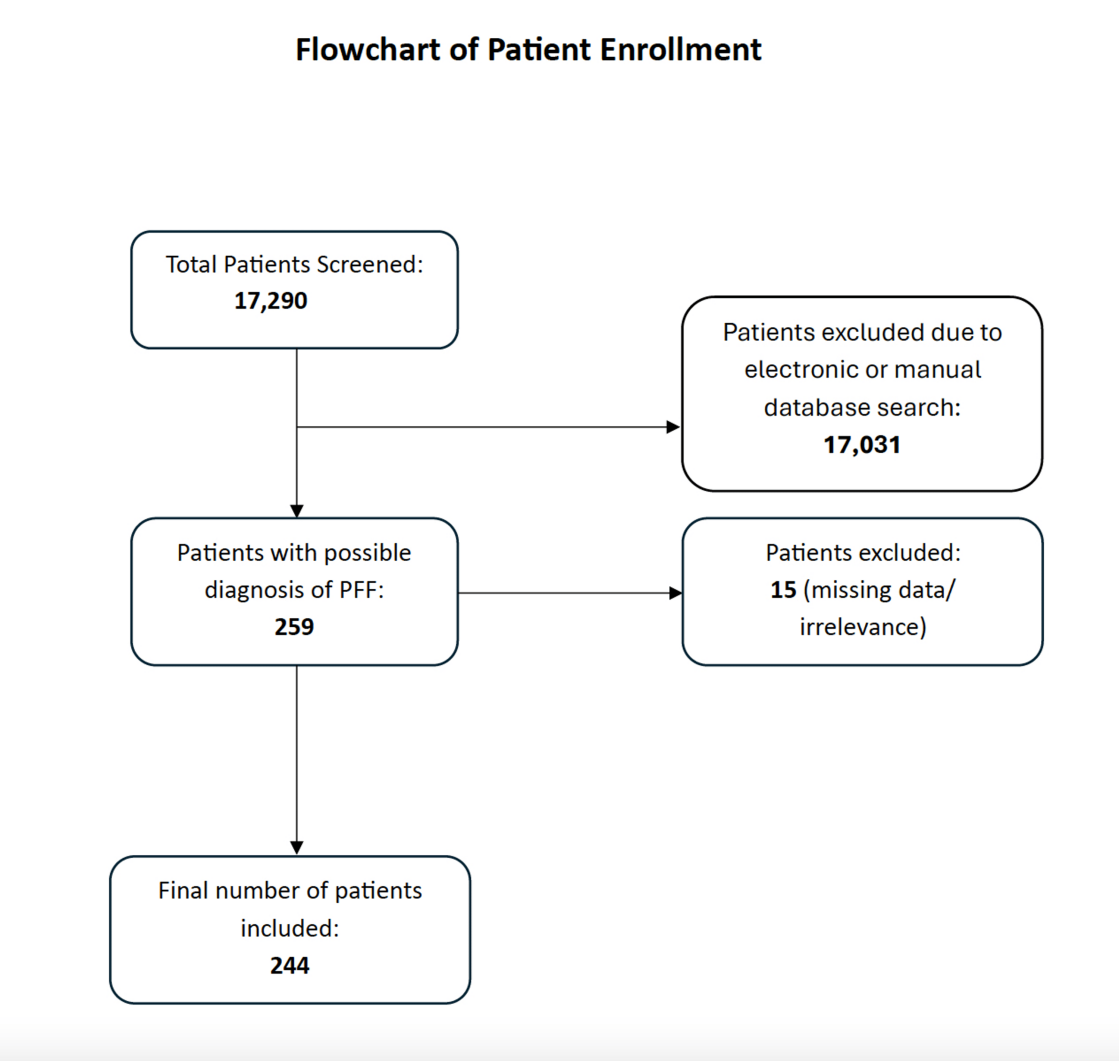
Flowchart of patient enrolment

The flowchart in Figure 1 illustrates the total number of patients initially screened, those excluded due to missing data or irrelevance, and the final count of patients included in the study. A total of 244 patients with PFFs were included in the study. The mean age and BMI were 78.2 years (11.09, 15-95) and 31.5 (4.07, 24-40.3), respectively. The demographic characteristics of the patients included in our study are listed in Table 1. The PFFs comprised 109 fractures around THAs, 73 around TKAs, 33 around femoral gamma nails, five between THA and TKA, and 24 involving different implants, such as THA and plates, dynamic hip screws or single plates. Among the THAs, there were 32 hybrid and 77 uncemented procedures, while all the TKAs were fully cemented. A significantly higher proportion of females, 211 (86.5%), compared to males, 33 (13.5%), was observed in the study population (χ^2^ test = 129.85, df = 1, p < 0.001).

**Table 1:** Baseline demographic characteristics of the study population *The values are given as raw numbers with the percentages in parentheses. ASA score: American Society of Anaesthesiologists score, PFF: periprosthetic femoral fracture, THA: total hip arthroplasty, TKA: total knee arthroplasty

Characteristic	Sub-characteristic	Value
Sex^*^	Female	211 (86.5)
	Male	33 (13.5)
ASA score^*^	1	10 (4.1)
	2	76 (31.1)
	3	122 (50)
	4	36 (14.8)
	5	0 (0)
PFF around^*^	THA cemented	32 (13.2)
	THA uncemented	77 (31.5)
	TKA fully cemented	73 (29.9)
	Femoral gamma nails	33 (13.5)
	Between THA and TKA	5 (2)
	Other implants	24 (9.9)

Number of PFFs

**Figure 2:**
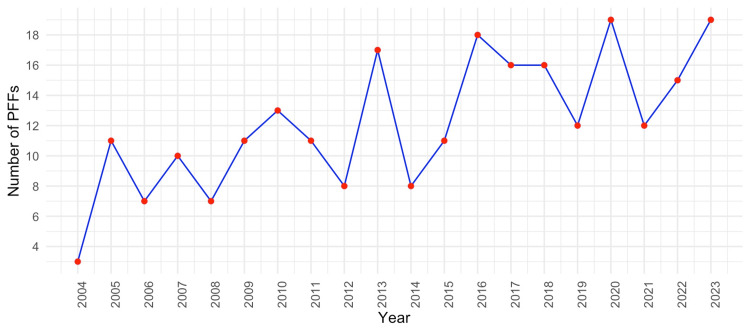
Illustration of the gradual increase in the mean annual admissions for periprosthetic femoral fractures (PFFs) at the orthopaedic department

**Figure 3:**
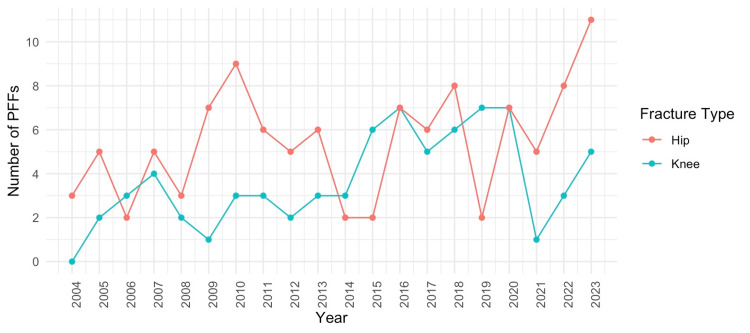
Incidence rates of periprosthetic femoral fractures (PFFs) around total hip arthroplasty (THA) and total knee arthroplasty (TKA) over time

Ninety-eight PFFs were recorded during the first decade of the study (2004-2013), compared to 146 PFFs recorded in the second decade (2014-2023). There was a significant increase in the number of PFFs between the first and second decade (Welch's t-test, mean: 9.8 vs. 14.6 cases per year, p = 0.01). Figure 2 illustrates the gradual increase in the mean number of PFFs admitted yearly at the orthopaedic department. There was no significant difference in the mean number of PFFs around THA between the two decades (Welch's t-test, 5.1 vs. 5.8 cases per year, p = 0.5539). However, there was a significant increase in the mean number of PFFs around TKAs between the first and second decade (Welch's t-test, 2.3 vs. 5.0 per year, p = 0.0027). Figure 3 shows the incidence rates of PFFs around THA and TKA over time. 

Age and gender characteristics of PFFs

**Figure 4:**
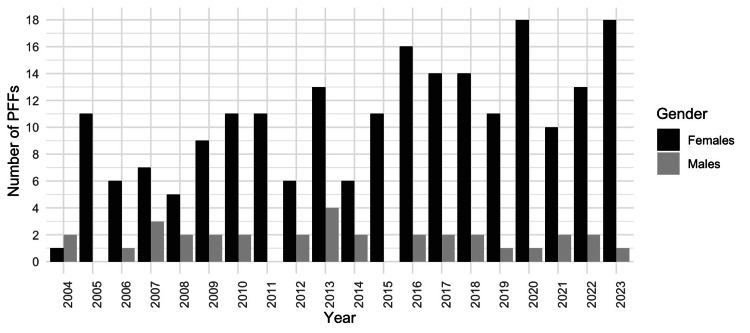
Differences in annual periprosthetic femoral fracture (PFF) admissions by gender during the study period

The average number of PFFs admitted to the hospital per year among females was significantly higher in the second than in the first decade. Specifically, the average annual number of PFFs for females rose from 8.0 in the first decade to 13.5 in the second decade (two-sample t-test, p = 0.0046, 95% CI: -9.08, -1.92). The mean number of PFFs per year for males remained relatively stable over the two decades (1.7 cases/year in the first decade and 1.6 cases/year in the second decade, Mann-Whitney U test, p = 0.89, 95% CI -0.81, 1.01). Figure 4 shows the differences by gender in PFF admissions per year in the hospital during the study period.

There was no statistically significant difference in the mean age between male and female patients (Welch's t-test, p = 0.07) during the whole study period. However, a notable age shift was observed between the two decades of study. The mean age of patients in the second decade was significantly higher than in the first decade (Welch's t-test, 75.60 vs. 79.60, p = 0.006). On the other hand, no significant age difference was found in the mean age of patients suffering from PFFs around THAs and TKAs (Welch’s t-test, 77.68 vs. 78.41, p = 0.61).

Τypes of PFFs

**Figure 5:**
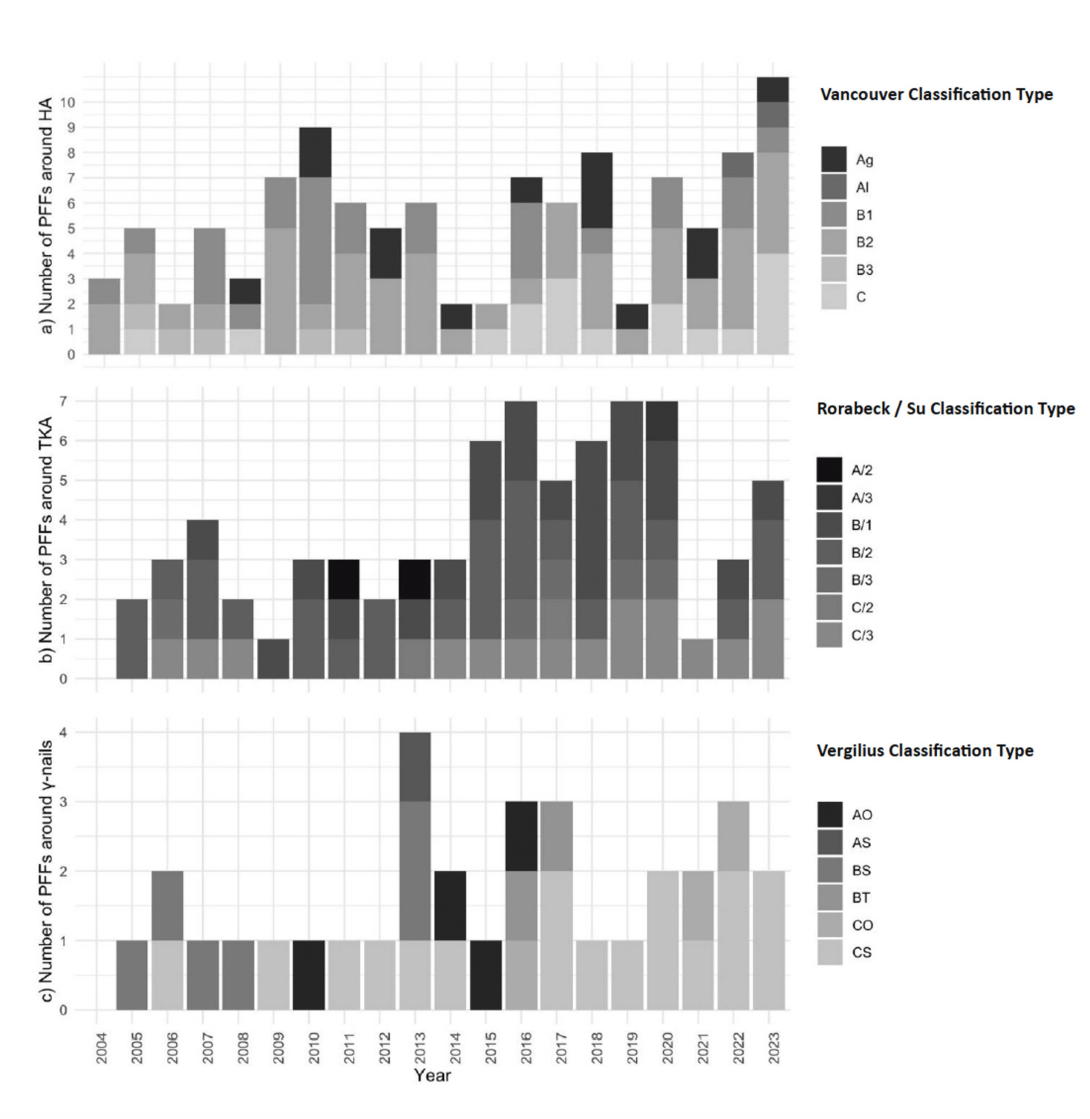
Annual distribution of PFFs around (a) total hip arthroplasties (THAs), (b) total knee arthroplasties (TKAs), (c) γ-nails

According to the Vancouver classification, the most prevalent type of PFF around THA was type B2 (41.2%), while around TKAs, it was the Rorabeck B/Su 2 category (35.6%). Table 2 demonstrates our study's number and percentage of PFFs based on the appropriate classification. Figure 5 illustrates the annual distribution of PFFs around THAs, TKAs, and γ-nails categorised by the relative classification throughout the study period.

**Table 2:** Distribution of periprosthetic femoral fractures (PFFs) categorised according to the relevant classification systems *The values are given as raw numbers with the percentages in parentheses. THA: total hip arthroplasty, TKA: total knee arthroplasty, γ-nails: gamma nails

Classification system	Type	Number (%)^*^
Vancouver classification (around THAs)	Ag	14 (12.9)
	Al	2 (1.8)
	B1	26 (23.9)
	B2	45 (41.2)
	B3	5 (4.6)
	C	17 (15.6)
Rorabeck/Su classification (around TKAs)	Rorabeck A/Su 2	2 (2.7)
	Rorabeck A/Su 3	1 (1.4)
	Rorabeck B/Su 1	22 (30.2)
	Rorabeck B/Su 2	26 (35.6)
	Rorabeck B/Su 3	4 (5.5)
	Rorabeck C/Su 2	2 (2.7)
	Rorabeck C/Su 2	16 (21.9)
Vergilius classification (around γ-nails)	AO	4 (12.2)
	AS	1 (3)
	BS	6 (18.2)
	BT	2 (6)
	CO	3 (9)
	CS	17 (51.6)
Pires classification (between THA and TKA)	IA	1 (20)
	IIA	2 (40)
	IIC	1 (20)
	IIIC	1 (20)

Management of PFFs 

Table 3 demonstrates the type of management per group of PFFs. The choice of treatment between ORIF and revision arthroplasty for treating PFFs around THAs remained consistent over the two decades. In the first decade, 25 cases were treated with ORIF and 17 with revision arthroplasty, while in the second decade, 28 cases were treated with ORIF and 18 with revision arthroplasty (Pearson's χ^2^ test = 0, p = 1). Concerning the choice of treatment between ORIF and revision arthroplasty for type B2 Vancouver PFFs, there were eight cases treated with ORIF and 12 with revision arthroplasty in the first decade, and three cases that were managed with ORIF and 17 with revision arthroplasty in the second decade. Although there was a trend towards the choice of revision arthroplasty in the second decade, this difference was not statistically significant (χ^2^ test = 2.0063, df = 1, p = 0.1567). Concerning the choice of treatment between ORIF (plate and screws) and revision arthroplasty for PFFs around TKAs, there were eight cases treated with ORIF and none with revision arthroplasty in the first decade, and 31 cases were managed with ORIF and three with revision arthroplasty in the second decade. This difference was insignificant (χ^2^ test = 0.011878, df = 1, p= 0.91). 

**Table 3:** Distribution of management type per classification system of periprosthetic femoral fracture (PFF) in two decades *The values are given as raw numbers. IF: internal fixation, ORIF: open reduction and internal fixation, PFF: periprosthetic femoral fracture, THA: total hip arthroplasty, TKA: total knee arthroplasty, γ-nails: gamma nails

Fracture type	Classification type	Type of management	Number^*^	
			First decade	Seconddecade
PFFs around THAs	Vancouver Ag	Conservative	3	7
		ORIF (plate/screws)	1	2
		Refused treatment	1	0
	Vancouver Al	Conservative	0	1
		Revision arthroplasty	0	1
	Vancouver B1	Conservative	2	1
		ORIF (plate/screws)	14	8
		Revision arthroplasty	1	0
	Vancouver B2	Conservative	2	1
		ORIF (plate/screws)	8	3
		Revision arthroplasty	12	17
		Refused treatment	0	2
	Vancouver B3	Conservative	1	0
		Revision arthroplasty	4	0
	Vancouver C	ORIF (plate/screws)	2	15
PFFs around TKAs	Rorabeck A/Su 2	Conservative	2	0
	Rorabeck A/Su 3	Conservative	0	1
	Rorabeck B/Su 1	Conservative	0	1
		IF retrograde nailing	1	2
		IF ORIF (plate/screws)	2	13
		External fixation (Illizarov)	1	0
		Refused treatment	1	1
	Rorabeck B/Su 2	IF retrograde nailing	6	3
		IF ORIF (plate/screws)	4	9
		External fixation (Illizarov)	1	1
		Revision arthroplasty	0	1
		Refused treatment	0	1
	Rorabeck B/Su 3	Conservative	0	1
		IF retrograde nailing	1	0
		IF ORIF (plate/screws)	0	1
		Refused treatment	0	1
	Rorabeck C/Su 2	IF ORIF (plate/screws)	1	1
	Rorabeck C/Su 3	Conservative	0	3
		IF retrograde nailing	2	1
		IF ORIF (plate/screws)	1	7
		Revision arthroplasty	0	2
PFFs around γ-nails	Vergilious AO	IF nailing	0	1
		IF ORIF (plate/screws)	1	1
		Refused treatment	0	1
	Vergilious AS	Refused treatment	1	0
	Vergilious BS	Conservative	1	0
		IF nailing	2	2
		External fixation	0	1
	Vergilious BT	IF nailing	0	1
		Refused treatment	0	1
	Vergilious CO	IF ORIF (plate/screws)	0	2
		Revision	0	1
	Vergilious CS	Conservative	0	1
		IF ORIF (plate/screws)	1	8
		IF nailing	3	3
		Refused treatment	0	1
Interprosthetic fractures (between THA and TKA)	Pires IA	ORIF (plate/screws)	0	1
	Pires IIA	ORIF (plate/screws)	1	1
	Pires IIC	ORIF (plate/screws)	0	1
	Pires IIIC	Conservative	0	1

The choice of treatment method (ORIF vs. revision arthroplasty) for PFFS around THAs was not significantly influenced by the age (Pearson's correlation coefficient= -0.149, p = 0.166) and ASA score (Fisher's exact test, p = 0.703) in our study. In the analysis of PFFs around TKAs, no significant correlation was found between patient age and the choice of treatment method (ORIF vs. revision arthroplasty) (Pearson’s correlation coefficient = 0.122, p = 0.441). The ASA score was also not significantly associated with the treatment method used (Fisher’s exact test, p = 0.725).

Length of hospital stay 

**Figure 6:**
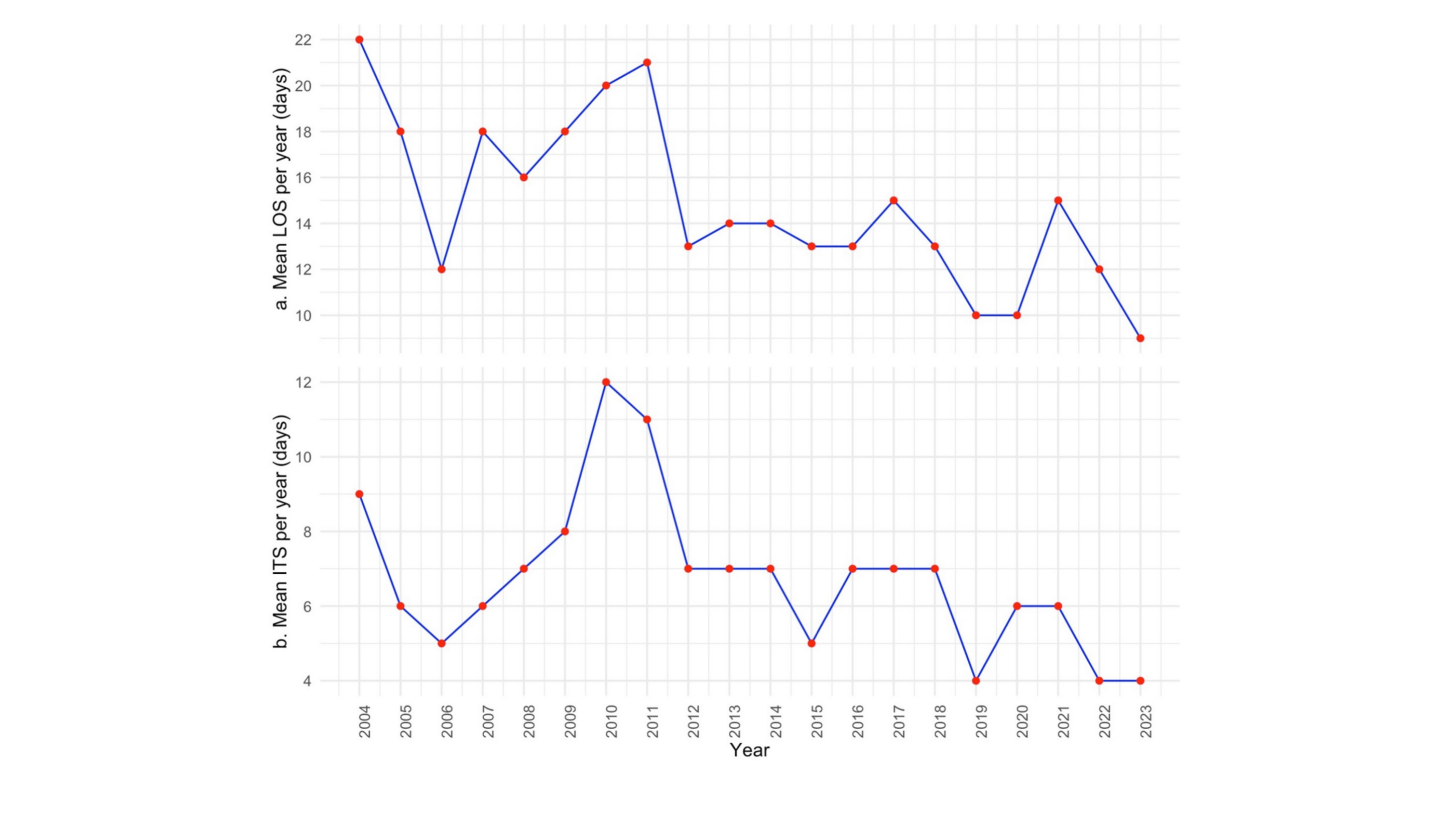
The figure depicts the (a) mean length of hospital stay (LOS) per year and (b) the mean time from admission to surgery during the study period

The average LOS significantly decreased from the first decade (17.2 days) to the second decade (12.2 days) (Mann-Whitney U test, p = 0.001). Figure 6a depicts the mean LOS per year, highlighting the consistent reduction over the study period. The mean LOS for patients who underwent ORIF decreased significantly from 18.21 days in the first decade to 15.88 days in the second decade (paired t-test, p = 0.021). Besides, patients undergoing revision arthroplasty for PFF also had a significant decrease in LOS from 26.25 days in the first decade to 15.81 days in the second decade (Wilcoxon signed-rank test, p = 0.032). 

The mean LOS was significantly longer for those patients treated with revision arthroplasty than ORIF during the first decade (Mann-Whitney U test, p = 0.008). However, in the second decade, LOS differences between the two treatment modalities were not significantly different (Mann-Whitney U test, p = 0.92). The LOS did not significantly differ between male and female patients (p = 0.33).

Time from admission to surgery 

The average time from admission to surgery was significantly shorter for the second decade (mean: eight vs. six days, Mann-Whitney U test, p = 0.02). The analysis of interval time to surgery did not vary significantly between men and women (Mann-Whitney U tests, p = 0.2648) or between those with PFFs around THAs or TKAs (Mann-Whitney U tests, p = 0.1157). Figure 6b depicts the mean time from admission to surgery, highlighting the consistent reduction over the study period.

## Discussion

Our research involves a retrospective analysis spanning 20 years, examining the occurrence of PFFs around THAs and TKAs in patients treated in a Greek academic orthopaedic department. Our study demonstrated a 50% increase in PFF incidence over the two decades. The significant increase in the incidence of PFFs during the second decade compared to the first decade was primarily associated with an increase in PFFs around TKAs rather than THAs. During the second decade, there was a higher frequency of PFFs in older female patients. Vancouver B2 PFFs were more commonly treated with revision arthroplasty during the second decade, while PFFs around TKAs were more frequently managed with ORIF using plate and screws or retrograde nails. During the study period, there was a substantial decrease in average LOS and the time from admission to surgery for patients with PFFs. 

Increased prevalence of PFFs

PFFs in our department have increased by 50% over the past decade. Recent studies have also shown an increase in PFFs, indicating a potential emerging public health concern and a possible new fracture epidemic [5]. Similar to the epidemic of hip fractures, the main risk factors include the ageing of the population, falls, and osteoporosis [5]. Furthermore, a significant contributing factor is the growing prevalence of THAs and TKAs in the Western population [2,4]. Matharu et al. estimated an increase of around 40% in THA and TKA volume in the United Kingdom by 2060 [11]. It seems that osteoporosis is the primary factor that can be influenced to decrease the incidence of PFFs. Osteoporosis is a prevalent condition among patients undergoing primary THA or TKA. Therefore, the arthroplasty surgeon must promptly identify and address it during the peri-operative period [12]. However, it frequently remains untreated [11,12]. In recent studies, the treatment rate for osteoporosis in patients scheduled to undergo THA or TKA is approximately 22.1 to 32.9% [13]. However, treatment strategies for this patient group are still debated [13]. 

Age

Our hospital's PFF rates and the average age of patients have increased over the past decade. The impact of the ageing of Western populations on healthcare systems, specifically on orthopaedic departments, has been well documented [14]. As we age, a higher risk of osteoporosis and increased susceptibility to falls due to declining physical and cognitive abilities can lead to a greater risk of fragility fractures in the femur. Elderly individuals with multiple health problems experience higher perioperative risks, leading to higher perioperative morbidity and mortality, increased LOS, and a higher rate of long-term complications. The economic impact of these fractures is significant and is anticipated to grow in the coming years [14]. It is essential to implement lifestyle modifications to mitigate the risk of fragility fractures and minimise the potential for falls. This includes engaging in weight-bearing exercises, ensuring a sufficient calcium and vitamin D intake, undergoing regular bone density assessments, evaluating the risk of falls, and providing appropriate treatment for osteoporosis [15].

Gender

Female patients significantly outnumbered male patients in the studied population, comprising 86.5% of the total participants. In addition, there was a significant rise in the incidence rate of PFFs among female patients over the two decades. Multiple studies have consistently shown that being female could be an independent risk factor for sustaining PFF [3]. The higher rise in PFFs in females than in males could be attributed to the higher prevalence of osteoporosis and longer life expectancy among women [16]. Although previously published data align with the predominance of females in similar patient groups, the discrepancy in distribution between the sexes was lower compared to our study. The single-centre retrospective study by Baggot et al. observed a 76% female preponderance [16]. Sershon et al. reported an even lower female preponderance of 58.3% [17].

Type of PFFs and management

Our study demonstrated an increased incidence of PFFs over time, suggesting a potential new fracture epidemic. Although the number of PFFs around THAs remained stable over the two decades, the number of PFFs around TKAs has increased. Interestingly, other studies support a stable incidence of hip and knee PFFs [5]. Despite affecting similar age groups, PFFs have distinct characteristics and unique fracture patterns compared to hip fractures [18]. According to Franklin and Malchau, prostheses and cement can create new points of weakness, known as stress risers, which are prone to fractures due to altered load distribution and increased mechanical stress [19]. Prostheses malalignment and loosening can also cause stress-riser effects on the cortex, leading to PFFs around THAs or TKAs. 

The complexity of PFF management often needs tailored treatment strategies involving a complex interplay of arthroplasty and orthopaedic trauma techniques [4]. The choice between ORIF and revision arthroplasty depends on factors like surgeon expertise and fracture characteristics. Managing PFFs requires either osteosynthesis around a well-fixed prosthesis or revision arthroplasty around a loose prosthesis [5]. Ensuring stability in these cases can be challenging, making managing PFFs increasingly nuanced. The decision of prosthesis stability preoperatively can be challenging, especially in type B2 PFFs around THAs and PFFs around TKAs when the femoral fracture involves the femoral implant [5]. Our study reveals that the choice between ORIF and revision arthroplasty for PFFs around hip prostheses has remained relatively stable, with THA revisions slightly increasing over the past two decades. Analysis of 60,887 lower extremity PFFs in the United States revealed stable incidence rates over time, with stable TKA revision rates and increasing THA revisions [20]. A recent study introduced the Total Knee Replacement Indication Scoring System (TKRISS), which provides a useful tool for assessing the potential indication for TKR in knee fractures, particularly in elderly patients [21]. 

Specifically for Vancouver type B2 PFFs, our data showed an increased use of revision arthroplasty in the second decade. This observation aligns with Joestl et al., who reported that 78% of Vancouver B2 PFFs were treated with revision arthroplasty, while 22% underwent ORIF with ORIF with a limited contact plate (LCP). Although ORIF with LCP can be effective, revision arthroplasty is generally preferred because of its superior stability, especially in cases involving femoral stem loosening [22]. González-Martín et al. also showed that 70% of patients with loose femoral stems underwent revision arthroplasty, while 30% received ORIF. They noted that while revision arthroplasty offers long-term benefits, ORIF provides advantages like shorter surgical times, fewer blood transfusions, and lower costs, which can be crucial for elderly patients with multiple comorbidities [23]. Gitajn et al. analysed 203 patients with Vancouver B PFFs and found that 17% were treated with ORIF, while 83% underwent revision arthroplasty. Although ORIF and revision arthroplasty offered similar short-term survival rates, revision arthroplasty provided better long-term outcomes [24].

Length of hospital stay and interval time to surgery

Our research revealed a significant decrease in the average LOS by five days and the mean interval time to surgery by two days over two decades, indicating improved efficiency in surgeons, hospital operations, and patient care for PFFs. Interestingly, the mean LOS of patients undergoing ORIF for PFFs was reduced by three days, and those undergoing revision surgery were reduced by more than 10 days between the first and the second decade. 

Our results align with trends observed in other Western populations, which also report reduced hospital stays and shorter times to surgery for PFFs. Baggott et al. found mean wait times of 2.5 days for PFF fixation and 4.5 days for revision surgery, with average LOS of 20.8 and 19.8 days, respectively [16]. A national observational study in England reported a median LOS of 14 nights and a total stay of 17 nights for PFFs [2]. Boddice et al. noted median delays of 5.9 days for revision vs. five days for ORIF in a retrospective single-centre PFF prevalence study, with stays of 17 days if surgery occurred within 72 hours, extending to 27 days if delayed [25]. Other studies reported shorter delays of days for those without inpatient mortality than for those with mortality [25], while others highlighted the importance of timely surgical intervention to reduce mortality and complications [26]. 

Over time, the reduced LOS of PFFs can be attributed to enhanced surgical expertise, including refined techniques and improved preoperative planning. Mondanelli et al. underscored that addressing the diverse challenges of PFFs, such as variability in stem designs and precise fracture fixation, requires advanced surgical skills and strategic planning, contributing to shorter LOS [27]. Furthermore, advancements in fracture classification and imaging enable more accurate diagnostics and timely interventions, thereby reducing the interval time to surgery [27]. Tsiridis et al. highlighted the role of contemporary graft substitutes, bone-inducing agents, and advanced implants in facilitating more efficient management of complex PFFs, further impacting hospital stay and surgical timing [28]. Besides, integrating geriatric care and addressing patient frailty improve overall outcomes, leading to reduced LOS and quicker time to surgery [29]. Despite improvements in PFF management, their LOS is substantially longer than patients managed for hip fractures [30], highlighting the remaining opportunity for further optimisation in reducing hospital stays and enhancing patient outcomes. 

The primary study's limitations include the documentation of fracture prevalence within a single tertiary care hospital, which limits generalizability to national and European trends. This study presents a 20-year dataset from an academic orthopaedic department in Southern Europe, featuring a predominantly Caucasian demographic and offering valuable insights. Notably, the study should have included a formal sample size calculation, which could affect the applicability of its findings. Moreover, the PFFs in our study were managed by orthopaedic surgeons of varying seniority, highlighting an inconsistent approach. The retrospective study nature and the absence of the M97.0 code in the electronic database search were acknowledged as limitations of our research. However, the number of missing cases was likely minimal, as a thorough manual search was undertaken to identify all relevant patients. 

## Conclusions

Our study emphasised the widespread occurrence of PFFs over the last two decades in a Greek orthopaedic referral centre. Our research findings indicate the rising prevalence of PFFs, particularly among women and older people. The treatment choice for PFFs has remained stable over the decades, influenced by clinician expertise and patient-specific factors. Revision arthroplasty was more commonly used for the management of Vancouver type B during the second decade. The enhanced surgical efficiency, hospital procedures, and patient treatment for PFFs led to a substantial decrease in LOS and time from admission to surgery during the study period.

This first documentation of PFFs in a Greek population demonstrates increasing incidence, particularly around TKAs, with improved hospital efficiency over time. These findings highlight the need for specialised PFF treatment centres and suggest the successful implementation of enhanced recovery protocols. Future studies should focus on identifying risk factors specific to the Greek population to develop preventive strategies.

## References

[1] Gibson A, Guest M, Taylor T, Harrold F, Gwynne Jones D (2024). The increasing complexity of femoral fragility fractures: incidence, fracture patterns and management over a 10-year period. Hip Int.

[2] Bottle A, Griffiths R, White S (2020). Periprosthetic fractures: the next fragility fracture epidemic? A national observational study. BMJ Open.

[3] Shichman I, Roof M, Askew N, Nherera L, Rozell JC, Seyler TM, Schwarzkopf R (2023). Projections and epidemiology of primary hip and knee arthroplasty in Medicare patients to 2040-2060. JB JS Open Access.

[4] COMPOSE Study Team (2022). Epidemiology and characteristics of femoral periprosthetic fractures : data from the characteristics, outcomes and management of periprosthetic fracture service evaluation (COMPOSE) cohort study. Bone Joint J.

[5] Tsiridis E, Haddad FS, Gie GA (2003). The management of periprosthetic femoral fractures around hip replacements. Injury.

[6] Brady OH, Garbuz DS, Masri BA, Duncan CP (2000). The reliability and validity of the Vancouver classification of femoral fractures after hip replacement. J Arthroplasty.

[7] Rorabeck CH, Taylor JW (1999). Periprosthetic fractures of the femur complicating total knee arthroplasty. Orthop Clin North Am.

[8] Su ET, DeWal H, Di Cesare PE (2004). Periprosthetic femoral fractures above total knee replacements. J Am Acad Orthop Surg.

[9] Toro G, Moretti A, Ambrosio D (2021). Fractures around trochanteric nails: the "Vergilius classification system". Adv Orthop.

[10] Pires RES, De Toledo Lourenço PRB, Labronici PJ, Da Rocha LR, Cavalcante FR, De Andrade MAP (2014). Interprosthetic femoral fractures: proposed new classification system and treatment algorithm. Injury.

[11] Matharu GS, Culliford DJ, Blom AW, Judge A (2022). Projections for primary hip and knee replacement surgery up to the year 2060: an analysis based on data from The National Joint Registry for England, Wales, Northern Ireland and the Isle of Man. Ann R Coll Surg Engl.

[12] James SJ, Mirza SB, Culliford DJ, Taylor PA, Carr AJ, Arden NK (2014). Baseline bone mineral density and boneturnover in pre-operative hip and knee arthroplasty patients. Bone Joint Res.

[13] Watanabe N, Miyatake K, Takada R (2022). The prevalence and treatment of osteoporosis in patients undergoing total hip arthroplasty and the levels of biochemical markers of bone turnover. Bone Joint Res.

[14] Liu E (2023). Hip fractures: mortality, economic burden, and organisational factors for improved patient outcomes. Lancet Healthy Longev.

[15] Dautzenberg L, Beglinger S, Tsokani S (2021). Interventions for preventing falls and fall-related fractures in community-dwelling older adults: a systematic review and network meta-analysis. J Am Geriatr Soc.

[16] Baggott PJ, Farook MZ, Pritchard M, Singh H, Bista A, Sobti A, Unnithan A (2022). Periprosthetic femoral fractures and their surgical outcomes between 2011 and 2021: a single-centre observational study. Cureus.

[17] Sershon RA, McDonald JF 3rd, Ho H, Hamilton WG (2021). Periprosthetic femur fracture risk: influenced by stem choice, not surgical approach. J Arthroplasty.

[18] Ramavath A, Lamb JN, Palan J, Pandit HG, Jain S (2020). Postoperative periprosthetic femoral fracture around total hip replacements: current concepts and clinical outcomes. EFORT Open Rev.

[19] Franklin J, Malchau H (2007). Risk factors for periprosthetic femoral fracture. Injury.

[20] Pagani NR, Varady NH, Chen AF, Rajaee SS, Kavolus JJ (2021). Nationwide analysis of lower extremity periprosthetic fractures. J Arthroplasty.

[21] Quattrini F, Andriollo L, Ciatti C, Maniscalco P, Benazzo F, Rossi SM (2024). Fractures around the knee in elderly patients: balancing fixation and arthroplasty approaches, a multicenter experience. Injury.

[22] Joestl J, Hofbauer M, Lang N, Tiefenboeck T, Hajdu S (2016). Locking compression plate versus revision-prosthesis for Vancouver type B2 periprosthetic femoral fractures after total hip arthroplasty. Injury.

[23] González-Martín D, Pais-Brito JL, González-Casamayor S, Guerra-Ferraz A, Ojeda-Jiménez J, Herrera-Pérez M (2022). Treatment algorithm in Vancouver B2 periprosthetic hip fractures: osteosynthesis vs revision arthroplasty. EFORT Open Rev.

[24] Gitajn IL, Heng M, Weaver MJ, Casemyr N, May C, Vrahas MS, Harris MB (2017). Mortality following surgical management of Vancouver B periprosthetic fractures. J Orthop Trauma.

[25] Boddice T, Harrison P, Anthony C, Ng AB (2023). Periprosthetic fractures around total hip replacement—is there a rush to fix?. J Clin Med.

[26] Wulbrand C, Füchtmeier B, Weber M, Eckstein C, Hanke A, Müller F (2024). Surgery within 24 hours reduces mortality and general complication rates in patients who have periprosthetic femoral fractures at the hip. J Arthroplasty.

[27] Mondanelli N, Troiano E, Facchini A (2022). Treatment algorithm of periprosthetic femoral fracturens. Geriatr Orthop Surg Rehabil.

[28] Tsiridis E, Narvani AA, Haddad FS, Timperley JA, Gie GA (2004). Impaction femoral allografting and cemented revision for periprosthetic femoral fractures. J Bone Joint Surg Br.

[29] PIPPAS Study Group (2024). Optimizing periprosthetic fracture management and in-hospital outcome: insights from the PIPPAS multicentric study of 1387 cases in Spain. J Orthop Traumatol.

[30] Konstantinou P, Kostretzis L, Fragkiadakis G (2024). Exploring quality of life and mortality in pertrochanteric fragility hip fractures in northern Greece: a single tertiary center study. J Clin Med.

